# The roles of migrasome in development

**DOI:** 10.1016/j.cellin.2023.100142

**Published:** 2023-11-28

**Authors:** Zhaocheng Zhai, Boqi Liu, Li Yu

**Affiliations:** The State Key Laboratory of Membrane Biology, Tsinghua University-Peking University Joint Center for Life Sciences, Beijing Frontier Research Center for Biological Structure, School of Life Sciences, Tsinghua University, Beijing, China

**Keywords:** Migrasome, Migrasome biogenesis, Development, Gastrulation, Angiogenesis

## Abstract

Migrasomes are newly identified vesicular structures that mainly come from the ends and crosspoints of retracting fibers in moving cells. Their creation is closely linked with cell movement and goes through three key steps: Nucleation, Maturation, and Expansion. They eventually get released in an event called migracytosis. Migrasomes have become an interesting focus in cell communication, especially during processes like development. They transport a mix of chemokines, growth factors, and morphogens. Their study can offer fresh perspectives on developmental gradients and improve our understanding of how development works. In the mini-review, we summarize our recent progress on the role of migrasomes in development, with a special focus on how migrasomes contribute to the spatial distribution of signalling molecules.

## 1 Migrasome discovery

By using transmission electron microscopy, A unique vesicular structure was first identified in the extracellular environment of normal rat kidney (NRK) cells *in vitro* in 2015 (Ma et al., 2015). These structure vesicles range from 0.5 μm to 3 μm in diameter and were described as pomegranate-like structures (PLSs) to feature their oval shape and highly variable number of smaller intraluminal vesicles within (Ma et al., 2015). Future observation by scanning electron microscopy and confocal microscopy further illustrated their attachment and sprouting from the retracting fibers (RFs) generated by NRK cells while migrating (Ma et al., 2015).

These findings also led us to the hypothesis that PLS formation might rely on cell migration. Therefore, we promoted the NRK cell migration by either culturing them on the fibronectin or knocking down (KD) the negative regulator of cell migration, *Sharpin* (Ma et al., 2015). Both methods accelerated cell migration speed and increased the number of PLSs generated. On the other hand, inhibiting the cell migration by drugs attenuated migration speed and reduced the formation number of PLSs (Ma et al., 2015). The later chemical screening experiments further revealed that drugs defecting cell migration can also reduce PLS number significantly (Lu et al., 2020), verifying the cell migration-dependent nature of PLS production. Thus, we decided to name them “migrasomes” to characterize their migration-dependent biogenesis (Ma et al., 2015). After the RFs of migrating cells are formed, the migrasomes begin to grow on fixed sites (Ma et al., 2015). The migrasome lifecycle starts with a rapid expansion followed by a stable period that its size remains constant until the RFs are broken due to cell migration (Ma et al., 2015).

Studying migrasomes heavily relies on imaging. In terms of markers for migrasome visualization, the transmembrane protein tetraspanin-4 (TSPAN4) has been identified as a marker. While TSPAN4 is localized on the plasma membrane, it is highly enriched on migrasomes (Ma et al., 2015) Additionally, integrin β1 is also highly concentrated in migrasomes and can serve as an imaging marker (Wu et al., 2017). Our previous research demonstrated that wheat-germ agglutinin (WGA) tagged with a fluorescent protein can also effectively label migrasomes both *in vivo* and *in vitro* (Chen et al., 2019).

Having established the imaging technique for migrasomes, it is worth noting that, in addition to their *in vitro* presence, migrasomes have been observed within various tissues *in vivo*. These observations span different organism models, including mice (Ma et al., 2015), chicks (Jiao et al., 2021), and zebrafish (Jiang et al., 2019). In mice, transmission electron microscopy has shown that migrasomes are especially abundant in cavities such as blood and lymph capillaries, as well as pulmonary alveoli (Ma et al., 2015). Furthermore, neutrophils generate a large number of migrasomes in blood vessels (Jiao et al., 2021). Migrasomes are also present in chicks and zebrafish during their developmental stages, playing a pivotal role in angiogenesis and organ morphogenesis (Jiang et al., 2019; Zhang et al., 2022).

## 2 Migrasome biogenesis

**Fig. 1:**
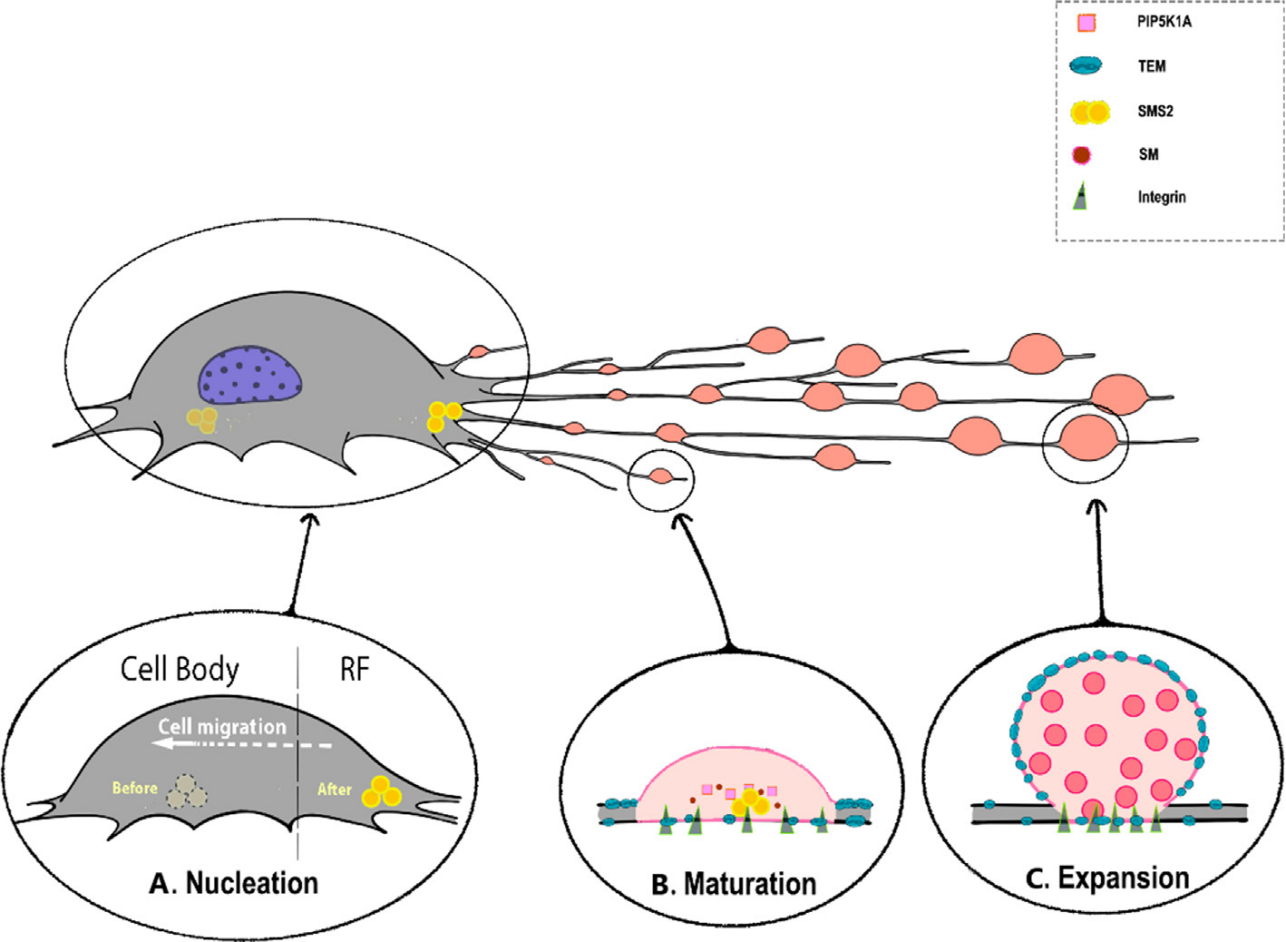
**The stages of migrasome biogenesis.** The migrasome formation can be roughly divided into three steps: **(A) Nucleation**, sphingomyelin synthase 2 (SMS2) will assemble into the SMS2 focus structure fixed on the basal membrane of the leading edge of the migrating cell body. As the cell migrates, SMS2 foci still remain immobile and will be left to the retracting fibers (RFs), which determine the migrasome formation sites. **(B) Maturation**, SMS2 converts the ceramide into the sphingomyelin (SM) which can further recruit the cholesterol. Meanwhile, the PIP5K1A also presents in the RFs and generates the phosphatidylinositol (4,5)-bisphosphate to activate the Rab35 that can recruit the integrin α5 to the bottom of migrasome formation sites. **(C) Expansion**, the migrasome start to expand and grow on the MFS, but the mechanism of the induction of swelling is still unclear, our biomimetic model suggests rapid increase in the membrane tension is sufficient to induce the swelling in a giant plasma-membrane vesicles. The tetranspanin-4 (TSPAN4) also forms tetraspanin-enriched microdomains (TEMs) with the cholesterol. During this stage, TSPAN4 primarily exists in the constricted regions on the RF tube instead of the swelling regions. The size of migrasome increases through translocation of the cytosolic contents, and more TEMs are recruited to the membrane of swelling region, which can be further assembled into the tetraspanin-enriched macrodomain (TEMA) to facilitate and stabilize the migrasome membrane.

The biogenesis of migrasome is still under intensive study, but currently we have already revealed several important steps for it, including the Nucleation, Maturation, and Expansion (Fig. 1).

### 2.1 Nucleation

Using living imaging, we found migrasome formation begins with the assembly of the sphingomyelin synthase 2 (SMS2) foci inside the migrating cells, which are located at the basal membrane of the leading edge (Fig. 1A) (Liang et al., 2023).

SMS2 is a transmembrane protein which catalyzes the conversion of ceramide to sphingomyelin (SM). Once the SMS2 foci form, they continue to grow but remain stationary as the cell migrates. These foci are left on the RFs and dictate the future migrasome formation sites (MFSs) (Liang et al., 2023). Both SMS2 and SM are critical for migrasome formation. Knocking down the gene for SMS2, *Sgms2*, or treating cells with SMS2 inhibitors significantly reduces the number of migrasomes (Liang et al., 2023). Additionally, we have shown that the SMS2 focus structure is crucial for migrasome formation. We produced an SMS2-S217A mutant that cannot form foci and observed that SMS2-S217A is evenly distributed on the cell base (Liang et al., 2023). This distribution leads to a marked decrease in migrasome number, even when exogenous SM is added in an attempt to rescue the situation (Liang et al., 2023). The formation of SMS2 foci represents the “Nucleation” step in migrasome formation, likely marking the beginning of migrasome biogenesis. Mechanistically, the *de novo* synthesis of SM mediated by SMS2 is essential for migrasome formation, possibly by maintaining migrasome structural integrity, enzymatically removing SM causes the migrasome to collapse rapidly.

### 2.2 Maturation

Once the position of MFS is decided, we found that the PIP5K1A will be recruited to MFSs in the early stage of MFSs (Fig. 1B) (Ding et al., 2023). PIP5K1A catalyzes the phosphatidylinositol (4,5)-bisphosphate (PI(4,5)P2) on the migrasome membrane (Ding et al., 2023). And knockout (KO) of the PI(4,5)P2 phosphatase can significantly increase the migrasome number (Ding et al., 2023). Mechanistically, PI(4,5)P2 contribute to migrasome formation by recruiting PI(4,5)P2-binding protein such as Rab35 (Ding et al., 2023). Indeed, the Rab35 is initially evenly spread along the RFs and will be recruited to migrasome formation sites later (Ding et al., 2023). Mutations in the binding site of Rab35 and PI(4,5)P2 stop Rab35 recruitment and impede the migrasome formation as well as the Rab35 KO (Ding et al., 2023).

Integrin α5 is subsequently recruited by the activated Rab35 to the migrasome formation sites (Ding et al., 2023). Knocking out Rab35 leads to a uniform and diffuse distribution pattern of integrin α5 on the RFs (Ding et al., 2023). Integrins accumulate at the bottom of migrasomes as heterodimers, each composed of one α and one β subunit (Wu et al., 2017). These integrin heterodimers can bind to their specific extracellular matrix (ECM) proteins, anchoring the migrasomes and tethering the RFs to the ECM (Wu et al., 2017). Ensuring the correct pairing of integrins with their respective ECM proteins is vital for migrasome formation (Wu et al., 2017). For instance, knocking down *Itga5* (the gene for integrin α5) impairs migrasome formation on fibronectin (the ECM protein for integrin α5β2), while the formation on other ECMs remains unaffected (Wu et al., 2017). Conversely, overexpressing *Itga3* (the gene for integrin α3) enhances migrasome formation on laminin 511 (the ECM protein for integrin α3β1), but not on other ECMs (Wu et al., 2017). Therefore, the successful recruitment of integrins is regarded as the key marker for MFS maturation.

### 2.3 Expansion

After migrasome maturation, migrasomes begin to grow and expand on the RFs (Fig. 1C). To understand how migrasome expansion occurs, a recent study utilized a biomimetic model based on the giant plasma-membrane vesicle (GPMV) generated from NRK cells (Dharan et al., 2023). This model allows researchers to simulate the RFs by drawing out a membrane tube from the aspirated GPMV (Dharan et al., 2023). They discovered that applying a sudden increase in membrane tension—achieved by rapidly raising the aspiration pressure on the membrane tube—led to pearling instability and the formation of migrasome-like structures in the tube (Dharan et al., 2023). This suggests that a change in membrane tension alone can induce initial migrasome-like swelling on the actual RFs. However, other mechanisms, yet to be identified, might also contribute to.

The initial expansion of migrasomes is stabilized by the recruitment of TSPANs. TSPANs are transmembrane proteins with four transmembrane domains. Previous research has shown that TSPAN often associates with cholesterol and other proteins to form tetraspanin-enriched microdomains (TEMs) (Huang et al., 2019). During the growth phase of migrasomes, TEMs can further assemble into a larger macrodomain (TEMA) in an SM-dependent manner (Huang et al., 2019; Liang et al., 2023). Although *Sgms2*-KD cells can recruit TSPAN4, they quickly lose it, leading to migrasomes shrinking back (Liang et al., 2023). Not surprisingly, the removal of either TSPAN4 or cholesterol also hinders the formation of migrasomes (Dharan et al., 2023).

The key role of TEMs in migrasome biogenesis was further demonstrate by *in vitro* reconstitution system which mimics the expansion phase of migrasome using the giant unilamellar vesicles (GUVs) (Huang et al., 2019). We noticed the migrasome-like structures are only able to form on the GUV membrane containing the TEMAs when the pulling force is induced to simulate the pull off the RFs during cell migration (Huang et al., 2019). Base on the data from the *in vitro* reconstitution system, a theoretical model for migrasome expansion is constructed, which suggest TEMA on the migrasome membrane actually obtain higher bending stiffness than their surrounding RF membrane, which results in the resistance of the MFS morphology change to form swellings while the overall RF is narrowed and flattened by the pulling force (Huang et al., 2019). The key prediction of this model, the elevated membrane bending rigidity on migrasome, is experimentally verified by atomic force microscopy (Huang et al., 2019). Indeed, our biomimetic model also shows the presence of TSPAN4 significantly prolongs the lifespan of the migrasome (Dharan et al., 2023). Altogether these results suggest that TEMA can both contribute to the growth and stabilization of migrasome through a physical mechanism.

## 3 Migrasome in early embryonic development

The positional information provides cues that determine the precise patterning of different organs during embryogenesis. This is established by the spatial and temporal distribution of morphogens. Morphogens are often secreted signals, and their dynamics are typically described by a simple diffusion model suggesting that the morphogens become progressively diluted as they diffuse between cells. However, there are scenarios that the classic diffusion models cannot explain, such as the diffusion of lipid-modified morphogens which are minimally diffusible, or the establishment of a gradient in an unenclosed environment (Stapornwongkul & Vincent, 2021). Our studies have shown that migrasomes can address these gaps in the current diffusion models, offering a new perspective on gradient establishment during development.

**Fig. 2:**
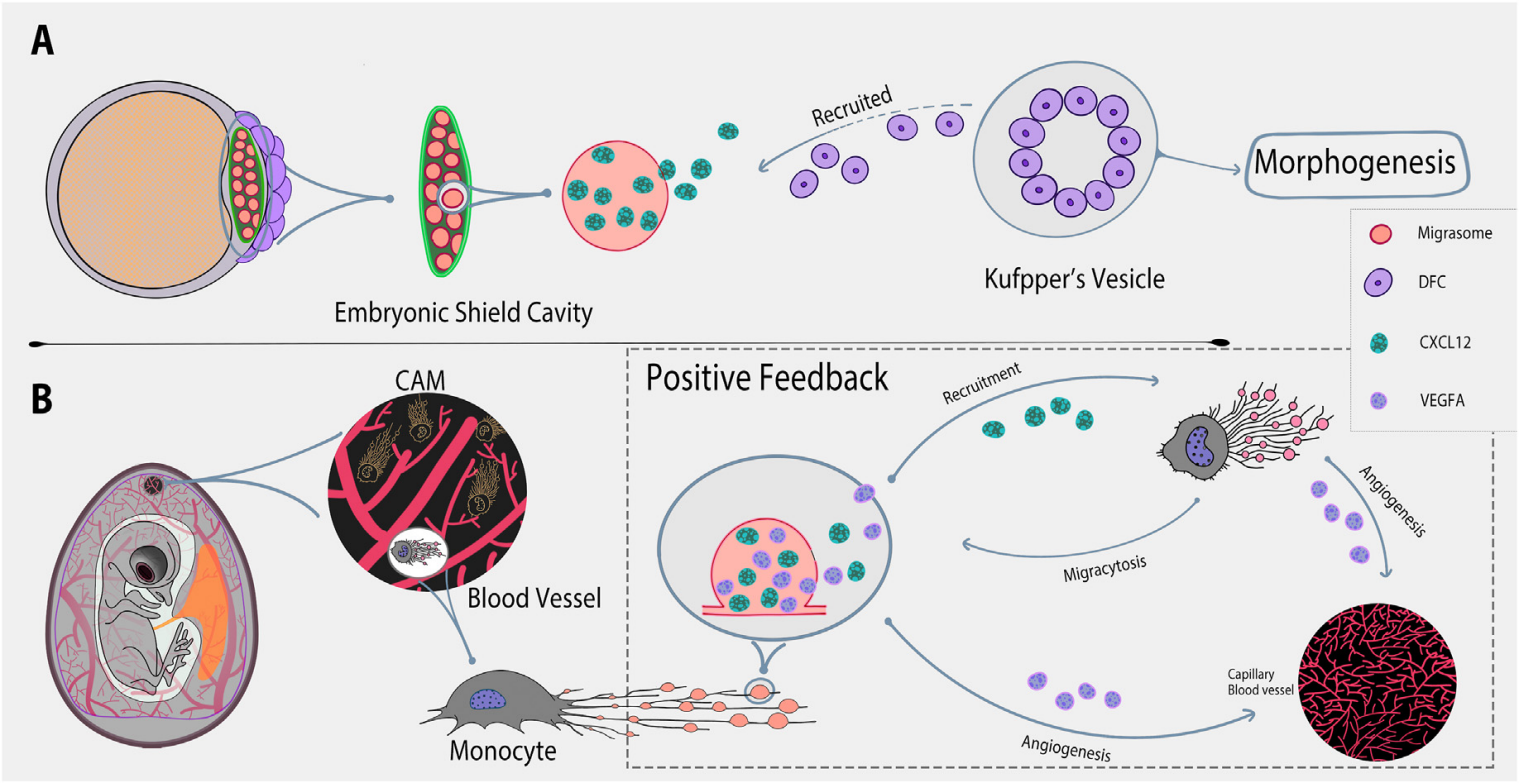
**The functions of migrasomes in zebrafish (A) and chick (B) development.(A)** The embryonic shield cavity between marginal mesendormal cells and yolk syncytial layer is enriched with the migrasomes which contains a large group of chemokines and morphogens and growth factors, especially the Cxcl12. Cxcl12 can recruit the dorsal forerunner cells (DFCs) making them clustered. DFCs will late form the Kupffer's vesicles (KVs) that build up the left-right body axis for the morphogenesis. **(B)** The chorioallantoic membrane (CAM) on day 9 of the chick embryo contain a large number of migrasomes generated by the monocytes from blood vessels. Their migrasomes carry the CXCL12 and VEGFA that can recruit more monocytes to CAM and promote the capillary angiogenesis, respectively. And the newly recruited monocytes presumably will release migrasomes containing CXCL12 and VEGFA again to form a positive feedback loop to promote capillary angiogenesis.

Our initial study of the migrasomes in development was undertaken in the zebrafish gastrulation owing to the conveniences of its transparent embryo and out-of-mother development (Fig. 2A) (Jiang et al., 2019). Gastrulation is a highly dynamic process driving the formation of three germ layers in triploblasts (ectoderm, mesoderm and endoderm), and their specification is achieved through morphogen gradient. There are also three stages involving the large scale of cell migration during gastrulation, i.e., Epiboly, Internalization and Convergent extension, covering nearly all the cell types in embryo. Therefore, gastrulation involves the establishment of many sophisticated morphogen gradients, the cells also demonstrate a vigorous migrating pattern, making the gastrulation stage a perfect guide for us to study the migrasomes in development.

During zebrafish development, beside the Tspan4a (homolog of TSPAN4 in zebrafish) and integrin β1b which were previously reported as the markers for migrasomes (Ma et al., 2015; Wu et al., 2017), the expression level of Tspan7 is also high based on our RNA-Seqencing and *in situ* hybridization results (Jiang et al., 2019). All of the *tspan4a*^−/−^, *tspan7*^−/−^, and *itgb1b*^−/−^ mutants demonstrate a severely reduced number of migrasomes during gastrulation (Jiang et al., 2019). Living imaging by transmission electron microscopy demonstrate large number of migrasome are generated during gastrulation stage of embryonic development by mesodermal and endodermal cells (Jiang et al., 2019). After detached form cells, migrasomes are re-distribute to various cavities in embryo, including a large cavity below the embryonic shield and dorsal forerunner cells (DFCs), which we named as the embryonic shield cavity, whereas maternal-zygotic (MZ) *tspan4a* or *tspan7* mutants demonstrated fewer migrasomes accumulated there (Jiang et al., 2019).

DFCs form Kupffer's vesicles (KVs) in zebrafish to establish the left-right body axis. MZ*tspan4a* and MZ*tspan7* mutants display abnormalities in their KV structure and impaired morphogenesis of left-right axis-related organs to varying extents. These defects can be rescued by exogenous migrasomes (Jiang et al., 2019). Interestingly, the exogenously injected migrasomes also gravitate spontaneously to the embryonic shield cavity (Jiang et al., 2019). The mechanism by which migrasomes accumulate in the embryonic shield cavity remains unclear. It is possible they are released by gastrula cells and then directed to the embryonic shield cavity by the movement of migrating cells during gastrulation, suggesting a potential mechanism for long-distance transport and the establishment of signalling gradients in development (Jiang et al., 2019). These results also underscore the capability of migrasomes to create spatially distributed patterns *in vivo*.

Mechanistically, our quantitative mass spectrometry revealed there is a wide range of chemokines, growth factors and morphogens presenting in the migrasomes during gastrulation, especially the Cxcl12 which is chemokine contributing to organ morphogenesis (Jiang et al., 2019). We confirmed the *cxcl12a* and *cxcr4b* (the receptor of Cxcl12a) mutants will demonstrate similar KV and laterality defects to what we observed in the MZ*tspan4a* and MZ*tspan7* mutants (Jiang et al., 2019). And we found that transplanting wild-type (WT) migrasomes to the *cxcl12* or *cxcr4b* mutants can rescue their defects as well (Jiang et al., 2019). Moreover, we found transplanting the beads embedded with WT migrasomes to the ventral side of the gastrula can drive the migration of the endosomal cells and DFCs to the ventral side, whereas the migrasomes purified from the *cxcl12a/b* morphants clearly attract fewer cells (Jiang et al., 2019). Together, this work shown migrasomes can establish a spatial pattern of Cxcl12 in the embryonic shield cavity for DFCs to support the development of zebrafish.

Chemical gradients from soluble ligands are recognized as primary signalling mechanisms in embryonic development (Ma et al., 2015). We postulate migracytosis as a novel developmental signalling mechanism where chemical signals are compartmentalized in membrane-bound structures and dispensed as unified entities. These signal units, potentially redistributed by embryonic fluid dynamics, might create localized signalling hubs in specific embryonic regions, such as the embryonic shield cavity. Our findings indicate a co-enrichment of chemokines, morphogens, cytokines, and growth factors within migrasomes. This implies the potential for multiplexed ligands to be encapsulated within a migrasome, transported to distinct sites, and concurrently released. This mechanism allows for sophisticated spatial and temporal signalling, crucial for the intricate orchestration of embryonic development.

## 4 The role of migrasome in embryonic angiogenesis

Similarly, we identified that migrasomes in chick development can also create a spatial pattern to induce the capillary angiogenesis on chorioallantoic membrane (CAM) (Fig. 2B) (Zhang et al., 2022). Previous research has already discovered a well-known mechanism for the angiogenic factor VEGF to form signal gradient by anchoring to the extracellular heparan sulfate proteoglycans. However, we also recognized a new mechanism to establish the VEGF gradient by migrasomes. In our research, we noticed that the monocytes can migrate out of the blood vessels and contribute to the majority of migrasomes on CAM on day 9 (CAM9D), removal of monocytes by either drugs or antibodies also reduces the number of migrasome along with the impairment of capillary formation without influencing the generation of large blood vessels (Zhang et al., 2022). We noticed the agarose embedded with exogenous migrasomes can successfully drive the migration, proliferation and sprouting of the endothelial cells (ECs) *ex vivo*, and treatment of migrasome *in vivo* promotes the EC tube formation and monocyte recruitment (Zhang et al., 2022). And KD or KO of the *TSPAN4* in CAM both greatly undermines monocyte migrasome generation, monocyte recruitment, capillary formation which can actually be rescued by transplanting the exogenous WT migrasomes (Zhang et al., 2022).

It is further confirmed by our quantitative mass-spectrometry that migrasomes purified from monocytes contain many angiogenetic factors or chemokines, including the VEGFA and CXCL12 which are known to play critical roles in capillary angiogenesis and monocyte recruitment during development, respectively (Zhang et al., 2022). Thus, the VEGFA and CXCL12 carried by the migrasomes can build a positive feedback loop to constantly enhance the capillary angiogenesis. Altogether, the migrasomes produced by monocytes from CAM contribute to capillary angiogenesis and monocyte recruitment by supplying the angiogenic factors and chemokines. This further suggests the migrasomes can precisely regulate the angiogenesis by delivering distinct signals that have complementary mechanisms. Therefore, migrasome seems to be a promising approach to learn more about the signal patterning during development.

It is important to distinguish between the roles of migrasomes in organ morphogenesis versus embryonic angiogenesis. In organ morphogenesis, migrasomes, produced by distal cells, are relocated to specific spatial locations, acting as remote “information packages” to establish local signalling centers. Conversely, in embryonic angiogenesis, migrasomes function akin to ant trail pheromones. Here, information-packed migrasomes are produced along the pathway, guiding capillary growth based on the direction of migrating monocytes.

## 5 Conclusion

In multicellular organisms, intercellular communication is crucial. As modern biology works to decipher how cells interact, our research on migrasomes in development provides a clear understanding of their function. Present data indicates that migrasomes play a central role in merging spatial, temporal, and chemical information. Beyond developmental biology, migrasomes may also be key in areas like immune responses, tissue regeneration, and metastatic processes. We believe that continued research on migrasomes will offer valuable insights across these fields.

## Authors’ contribution

Zhaocheng Zhai: Writing – Original Draft; Li Yu and Boqi Liu: Writing – Reviewing and Editing; Li Yu: Supervision. All the authors read and approved the final manuscript.

## Declaration of competing interest

The authors declare that they have no known competing financial interests or personal relationships that could have appeared to influence the work reported in this paper.
